# Atmospheric carbon monoxide and hospitalization for mental and behavioral disorders: insights from a longitudinal study in Shijiazhuang

**DOI:** 10.3389/fpsyg.2025.1573556

**Published:** 2025-04-30

**Authors:** Peihua Hu, Wenting Lu, Xian Gao, Yating Li, Yanli Yang, Wanyi Yin, Liang Dong, Ruojia Ren, Xueyi Wang

**Affiliations:** ^1^Institute of Mental Health, First Hospital of Hebei Medical University, Shijiazhuang, China; ^2^Hebei Medical University and Hebei Technical Innovation Center for Mental Health Assessment and Intervention, Hebei Clinical Research Center for Mental Disorders and Institute of Mental Health, Department of Psychiatry, The First Hospital of Hebei Medical University, Shijiazhuang, China; ^3^Department of Clinical Pharmacy, The First Hospital of Hebei Medical University, Shijiazhuang, China; ^4^Department of Nursing, The First Hospital of Hebei Medical University, Shijiazhuang, China; ^5^Department of Nephrology, The First Hospital of Hebei Medical University, Shijiazhuang, China; ^6^Department of Hematology, The First Hospital of Hebei Medical University, Shijiazhuang, China; ^7^Department of Hospital Infection Management, The First Hospital of Hebei Medical University, Shijiazhuang, China

**Keywords:** carbon monoxide, air pollution, mental health, hospitalization, MAPK3, behavioral disorders, epidemiology

## Abstract

**Background and aim:**

Carbon monoxide (CO), a prevalent environmental pollutant, has been implicated in adverse mental health outcomes, but the mechanistic relationship between atmospheric CO levels and hospital admissions for mental and behavioral disorders remains unclear. This study investigates the epidemiological link between atmospheric CO and hospitalizations for mental health conditions in Shijiazhuang, China.

**Methodology:**

Clinical data from patients hospitalized with mental and behavioral disorders at The First Hospital of Hebei Medical University between January 2014 and December 2020 were analyzed. Daily atmospheric CO levels, temperature, and relative humidity were concurrently monitored. A generalized additive model (GAM) was used to explore the correlation between CO levels and hospital admissions. Blood samples from patients with depressive disorders were analyzed for MAPK3 expression, and a mouse model of CO-induced depression was employed to further explore the molecular mechanisms.

**Results:**

A total of 15,890 hospitalization records were included. A significant positive correlation was identified between CO levels and the number of daily hospitalizations, with the strongest effects observed when CO concentrations exceeded 40 μg/m^3^. This association was most pronounced in males and individuals aged over 45, as well as during both warm and cold seasons. A two-pollutant model confirmed CO as a major factor affecting hospitalizations, independent of other pollutants like nitric oxide and sulfur dioxide. Additionally, elevated MAPK3 expression was found in the blood samples of depressed patients, and treatment with the MAPK inhibitor PD98059 alleviated CO-induced depression in a mouse model.

**Conclusion:**

This study provides compelling evidence for a significant association between atmospheric CO and hospitalizations for mental and behavioral disorders. The findings suggest that CO exposure may exacerbate mental health conditions, particularly in vulnerable populations. These insights underline the importance of air quality management and highlight potential pathways for therapeutic interventions targeting CO-induced mental health disorders.

## Introduction

As the frequency and intensity of severe air pollution events continue to increase in various regions around the world, the impact of air pollution on mental disorders has emerged as a matter of growing concern. Indeed, there are accumulated studies suggesting that air pollution may increase the risk of suicide, depression, autism, and anxiety during pregnancy (Bakian et al., 2015; Wang et al., 2018; Lin et al., 2017; Volk et al., 2013). The mechanistic link between air pollution and mental disorders remains to be fully elucidated, despite oxidized pollutants being suggested to be involved in the pathogenesis of depression (Guan et al., 2015). Carbon monoxide (CO), a commonly detected air pollutant resulting from incomplete organic combustion, is toxic by inhalation at high concentrations (Xu et al., 2020). CO binds to hemoglobin with high affinity, impairing oxygen delivery to tissues, leading to hypoxia. This hypoxic state triggers oxidative stress and lipid peroxidation in the central nervous system, resulting in neuronal injury. Additionally, CO exposure induces the release of excitatory amino acids like glutamate, causing excitotoxicity and apoptosis (Ho et al., 2012). These mechanisms collectively contribute to neuropsychiatric sequelae, including cognitive deficits, mood disorders, and delayed neuropsychiatric effects (Watt et al., 2018).

Szyszkowicz et al. indicated that atmospheric pollutants such as sulfur dioxide and CO would be associated with increases in the number of depression diagnoses in psychiatric emergency rooms, implicating the important role of CO in mental disorders (Szyszkowicz et al., 2016). More recently, the pro-oxidant constituents of vehicle exhaust including CO were demonstrated to cause memory deficits and anxiety and depression-like behaviors, which were accompanied with elevated levels of serum corticosterone and markers of oxidative stress and inflammatory markers in male rats (Kochi et al., 2021). Pollutants are also believed to impair normal cellular functions by affecting mitochondria (Kochi et al., 2023). Despite these reported adverse effects of CO on nervous system and so cognitive behaviors, on the other hand, there are also studies suggesting that CO might be a neuroprotective substance that can reduce inflammation, mediating neurotransmission and regulating hemodynamics in the brain (Hanafy et al., 2013). CO is suggested to act as a signaling molecule that promotes cellular survival pathways (Mahan, 2012). Besides, CO was shown to reduce oxidative stress and apoptosis in neuronal cells, thereby protecting against ischemic brain injuries (Guo et al., 2024). Additionally, CO’s ability to enhance neurogenesis and improve cognitive functions has been observed in animal models (Watt et al., 2018). An exogenous administration of CO was also found able to block LPS-induced depressive- and anxiety-like behaviors in mice (Luo et al., 2021). The impact of CO on mental disorders is therefore not entirely consistent.

The mechanistic links between exposure to environmental pollutants with severe mental disorders have been investigated in different studies. The hypothalamic–pituitary–adrenal axis, which regulates stress responses, has been identified as a key pathway that is often disrupted by pollutants including CO (Thomson et al., 2019). Pollutant exposure can affect neurotransmitter systems like the serotoninergic system, leading to imbalances that contribute to mood disorders (Thomson et al., 2019). Additionally, the mitogen-activated protein kinase (MAPK) was demonstrated to play a key role in modulating neuronal survival, synaptic plasticity, and neuroinflammation upon pollutant exposure (Ijomone et al., 2021). The studies have indicated the complex interplay between environmental pollutants and mental health, emphasizing the need for further research to develop effective preventive measurement and therapeutic intervention (Catapano et al., 2024).

In view of the debated role of CO in mental disorders, the present longitudinal study was aimed to explore the impact of atmospheric CO on the number of hospitalizations for mental disorders in Shijiazhuang City of Hebei Province of the People’s Republic of China. Shijiazhuang City was selected as the research location because the atmospheric CO has been increasing due to the rapid development of transportation and industrial and mining enterprises over the past few years. Being one of the important central cities in the Beijing-Tianjin-Hebei region having a permanent population of 11.2 million and an urbanization rate of 71.4%, findings from Shijiazhuang City would provide valuable insights into the relationship between CO and mental disorders in populated cities around the world. At the same time, with an initial hope to identify new therapeutic target for treatment of CO-induced depression and given the potential role of MAPK in such disorder, the present study also determined the blood MAPK3 transcript among patients with depression and characterized the role of MAPK3 in a mouse model of CO-induced depression.

## Methodology

### Clinical data collection

Clinical records of patients who were hospitalized for mental and behavioral disorders into The First Hospital of Hebei Medical University from January 2014 to December 2020 were retrieved. There is strong patient adhesion in Shijiazhuang and the surrounding areas, with most hospitalized patients coming from Shijiazhuang and its vicinity. The disorders were defined according to the International Classification of Diseases (10th edition, ICD-10) with the code F01-F99. Data were stratified into subgroups of patients’ age (i.e., < 45 and ≥45 years), gender (i.e., male and female) and season of hospitalization (i.e., cold [October to April] and warm [May to September]). The study was approved by the Ethics Committee of First Hospital of Hebei Medical University (IRB approval number: 20190592) and the requirement for informed consent was waived because the review of the patient data was retrospective and anonymous.

### Air pollution and meteorological data collection

Atmospheric levels of pollutants including CO were retrieved from the website of the Ministry of Ecology and Environment of the State Council of the People’s Republic of China. This data was collected real-time from seven fixed stations in the urban areas of Shijiazhuang City. The stations are far away from main roads, industrial sources, residential areas, and emissions from burning coal, oil, or waste; therefore, the monitored results would truly reflect urban air pollution levels rather than local pollution sources. Daily meteorological data like mean temperature (MT) and relative humidity (RH) were also obtained from the National Meteorological Information Centre of China Meteorological Data Service Centre.

### Determination of blood MAPK3 mRNA

RNA was extracted from whole blood samples collected from patients with depression as well as healthy individuals for first-strand cDNA synthesis. MAPK3 and GAPDH were then amplified using real-time PCR. The relative level of MAPK3 was calculated using 2^-∆∆CT^ approach. This technique begins by determining the CT (threshold cycle) values, which indicate the cycle number at which the PCR product becomes detectable; lower CT values correspond to higher gene expression levels. The first step involves calculating the ∆CT for each sample by subtracting the CT value of a reference (housekeeping) gene from that of the target gene. Next, to assess the relative expression between experimental and control groups, the ∆∆CT is calculated by finding the difference in ∆CT values between these groups. Finally, the relative expression level of the target gene is derived using the formula 2^(−∆∆CT).

### Mouse model of CO-induced depression

To further decipher the molecular pathogenesis of CO-induced mental disorders, the functional role of MAPK3 and its associated pathway activation was studied in mice with depression induced by CO exposure. C57BL/6J mice, aged 6–8 weeks with initial weight upon arrival was 18–20 grams, were purchased (Henan Skobes Biotechnology, Henan, China). The active and exploratory behavior of this mouse strain makes them a good model for behavioral studies. The mice were housed at a constant temperature of 23 ± 2°C and subjected to a 12-h light/dark cycle (lights on from 8 AM to 8 PM), with free access to food and water. After a two-week acclimatization period, the mice were randomly divided into four groups: Control group (*n* = 6), CO-induced depression model (i.e., CO, *n* = 6), Control group + MAPK3 inhibition group (i.e., Control + PD98059, *n* = 6), and CO-mediated depression model + MAPK3 inhibition group (i.e., CO + PD98059, *n* = 6). PD98059 is a selective potent inhibitor to MAPK3.

Depression was induced in mice by exposing mice to a 3,000 ppm CO environment for 40 min using a static inhalation exposure method. This exposure condition would ensure robust and uniform exposure of mice to CO. After CO exposure, the mice received injections of saline, as the negative control, to assess the effects of CO alone on their behavior. The mice would exhibit corresponding depressive behaviors, such as anhedonia, despair, significant reduction in appetite, social withdrawal, and cognitive impairment. PD98059 was prepared as solution (10 mg/ml, dimethyl sulfoxide [DMSO]) and was injected intraperitoneally at a dosage of 10 mg/kg.

The effect of MAKP3 treatment on CO-inflicted mice was studied by examining their behaviors by different tests including sucrose preference test (SPT), open field test (OFT), and Y-maze test. After the tests, mice were humanly sacrificed. Protein levels of brain-derived neurotrophic factor (BDNF) and MAPK3 in brain tissues were then examined using western blotting.

### Statistical analysis

To investigate the effect of individuals’ age and gender and season on the occurrence of mental and behavioral disorders, data were stratified into subgroups for in-depth analysis. The time series regression method was used to study the acute impact of particulate matter (i.e., CO) on the hospitalization of patients with mental and behavioral disorders. Given that data about patients’ hospitalization days usually follow a quasi-Poisson distribution, the over dispersed generalized additive model (GAM) was used to analyze the relationship between CO and hospitalization days in patients with mental and behavioral disorders. To control for potential confounding effects, several covariates were introduced, including (i) natural cubic regression as a smooth function of calendar time with 7 degrees of freedom (df) per year, excluding unmeasured long-term and seasonal trends beyond 2 months; (ii) natural smooth functions of average temperature (6, df) and relative humidity (3, df) to control weather conditions affected by nonlinear confounding effect; and (iii) any indicator variables representing “day of the week” and public holidays.

The main model was described as follows: logE (Yt) = βzt + ns (time, df) + ns (temperature, 6) + ns (humidity, 3) + dow + intercept, where e (yt) represents the number of hospitalizations for mental behavioral disorders on day t expected number of people; β represents the logarithmic correlation rate of admissions for mental and behavioral disorders associated with a unit increase in particulate matter pollutants; zt represents the pollutant concentration on day t; dow represents a dummy variable for the day of the week; ns represents the natural cubic regression smoothing function.

After the basic model was established, we further introduced single-day lag 0–7 and multi-day moving average exposure, including lag0-1, 0–2, 0–3, 0–4, 0–5, 0–6. By adding three df natural spline functions to the model, the exposure-response relationship curve between CO and hospitalization for mental behavioral disorders was drawn.

To test the stability of the model, a sensitivity analysis was performed. Second, we built a two-pollutant model to examine the robustness of effect estimates after adjusting for co-pollutants.

In addition, we conducted three levels of analysis to explore potential effects by gender, age (<45 years and ≥45 years) and season (warm: April to September, cold: October and January to April). We further tested the statistical significance of the differences between estimates of formation effects by calculating 95% confidence intervals (CI) using the following equation.


$$95\%CI=\left({\hat{Q\;}}_{1}-{\hat{Q\;}}_{2}\right)\pm 1.96\sqrt{\hat{{se}_{1}^{2}}+\hat{{se}_{1}^{2}}}$$

Where and are the two class estimates, and are their respective standard errors.

The statistical test was a two-sided test, and *p* < 0.05 was defined as a statistically significant difference. All statistical models were run in R software (version 3.3.3) using the “MGCV” package. The change rate and 95% CI of mental and behavioral disorders caused by every 10 μg/m^3^ increase in CO were used as indicators.

For the determination of MAPK3 transcript in patients’ blood samples and mouse model of CO-induced depression, the comparison was done using non-parametric tests.

## Results

### Cohort characteristics

**Figure 1:**
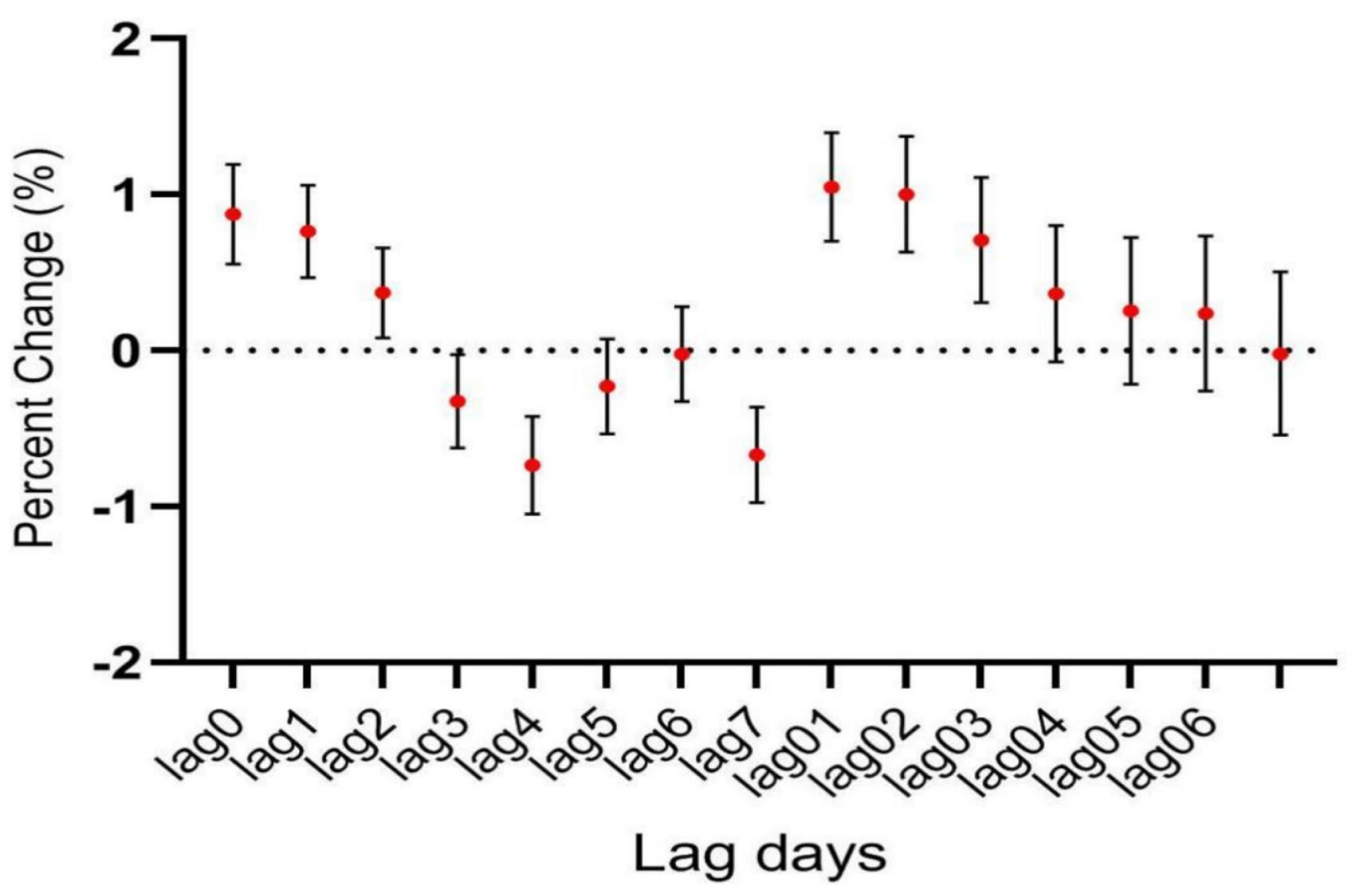
The relationship between atmospheric CO concentration and hospitalization for mental and behavioral disorders.

**Figure 2:**
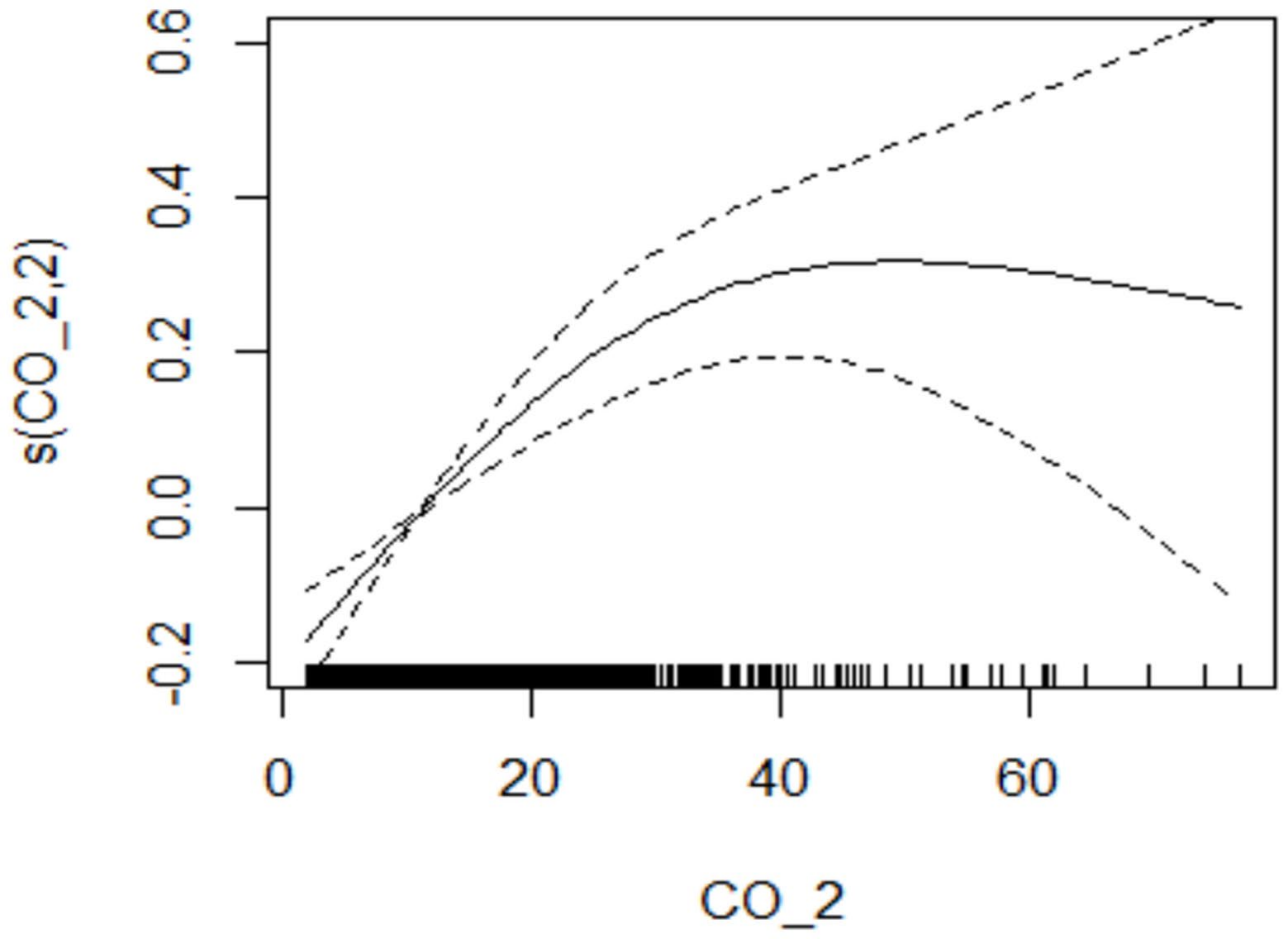
The relationship between atmospheric CO concentration and hospitalization for mental and behavioral disorders at lag02.

During the study period, there were a total of 15,890 cases of hospitalization for mental and behavioral disorders in Shijiazhuang City. The majority was male (*n* = 10,083), and there were 9,143 cases being aged <45 years. Slightly more cases were seen in cold than in warm seasons (cold season, 8,378 cases; warm season, 7,512 cases). The daily average concentration of CO was 45.4 μg/m^3^, with the daily average temperature and relative humidity as 15 degree and 56%, respectively (Table 1). Results showed that there was a significant lag effect of CO, with lagging for 2 days (i.e., lag02) being the maximum estimated value (Figure 1). A E-R with positive correlation between lag02 and daily number of hospitalizations was seen, in which a peak was achieved when the concentration of CO was greater than 40 μg/m^3^ (Figure 2).

**Table 1:** Summary of descriptive statistics during the study period.

Variable	25th Percentile	MEDIAN	75th Percentile
CO (μg/m^3^)	31.7	45.4	63.2
Total	2	3	6
Gender			
Male	0	0	2
Female	1	3	5
Age			
<45	1	2	4
≥45	1	1	3
Season			
Warm	2	3	6
Cool	2	3	7

### Effect of individuals’ age and gender and season

**Figure 3:**
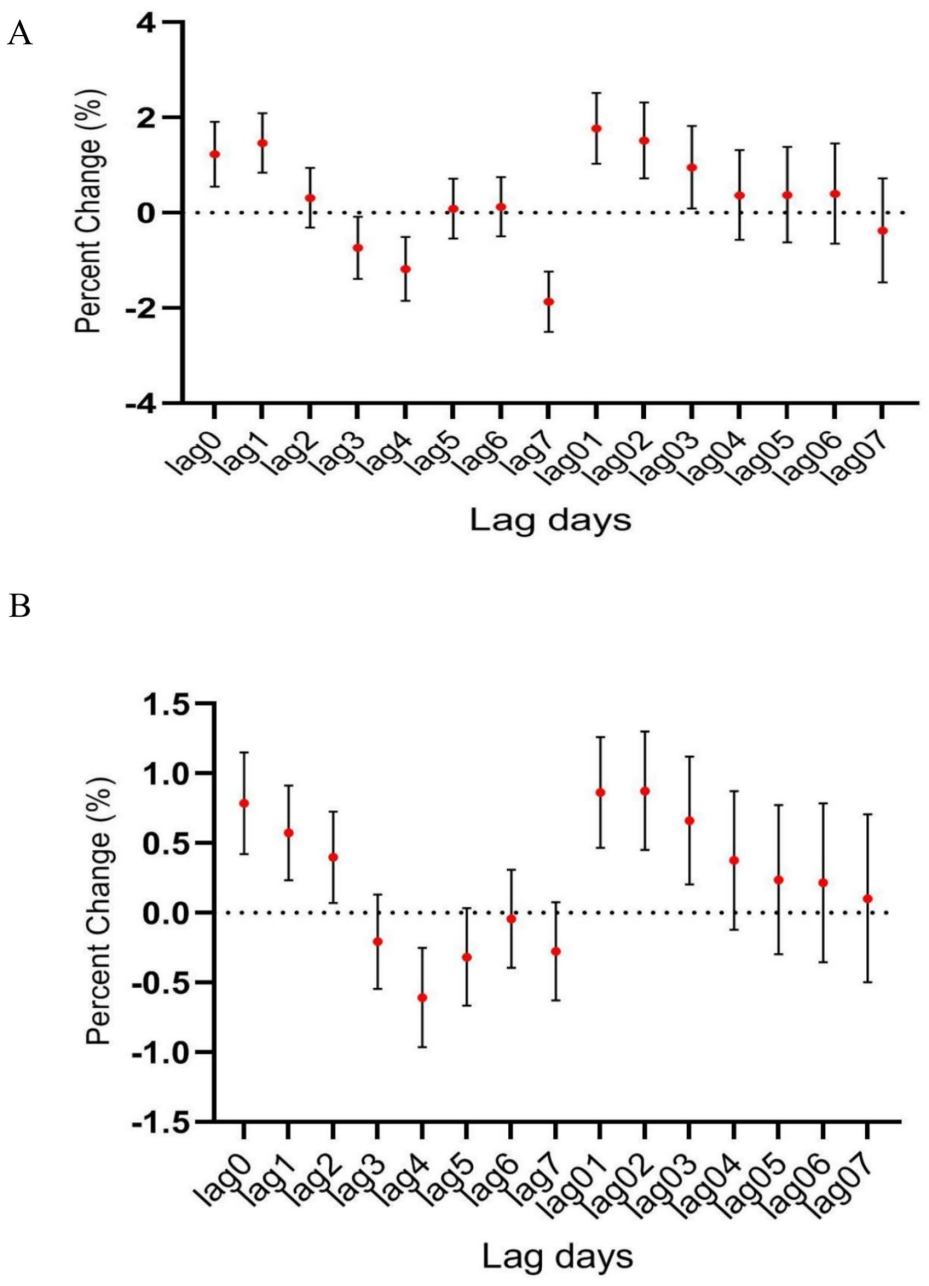
The relationship between atmospheric CO concentration and hospitalization for mental and behavioral disorders of **(A)** male and **(B)** female patients.

In male patients, CO was significantly associated with hospitalization for psychiatric and behavioral disorders (OR, 1.336; 95% CI, 0.041–1.972) (Figure 3A), however, such positive correlation was not seen in female patients despite there was slight increase in female patient hospitalizations along with the increasing CO level (Figure 3B).

**Figure 4:**
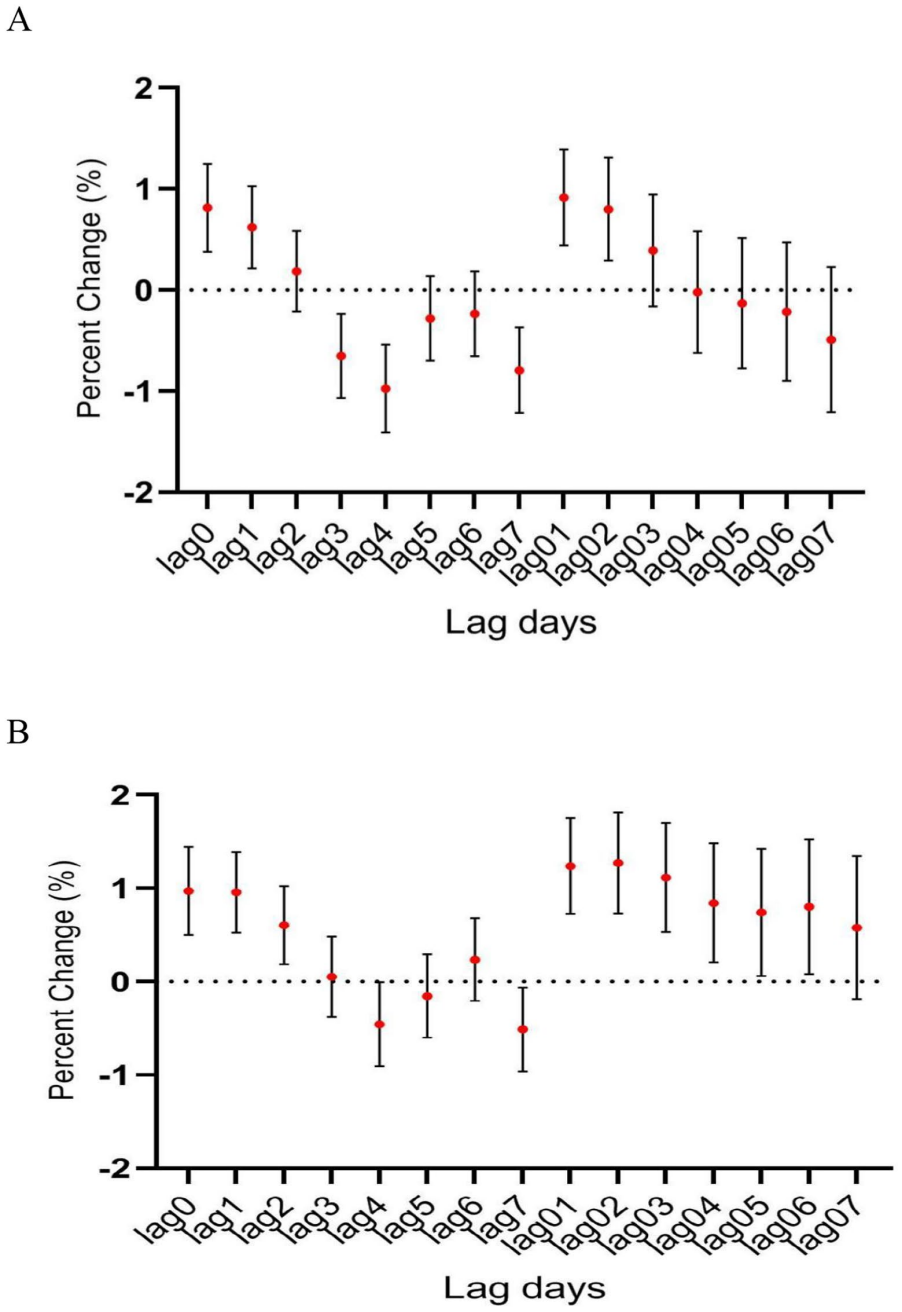
The relationship between atmospheric CO concentration and hospitalization for mental and behavioral disorders of patients aged **(A)** <45 years and **(B)** ≥ 45 years.

**Figure 5:**
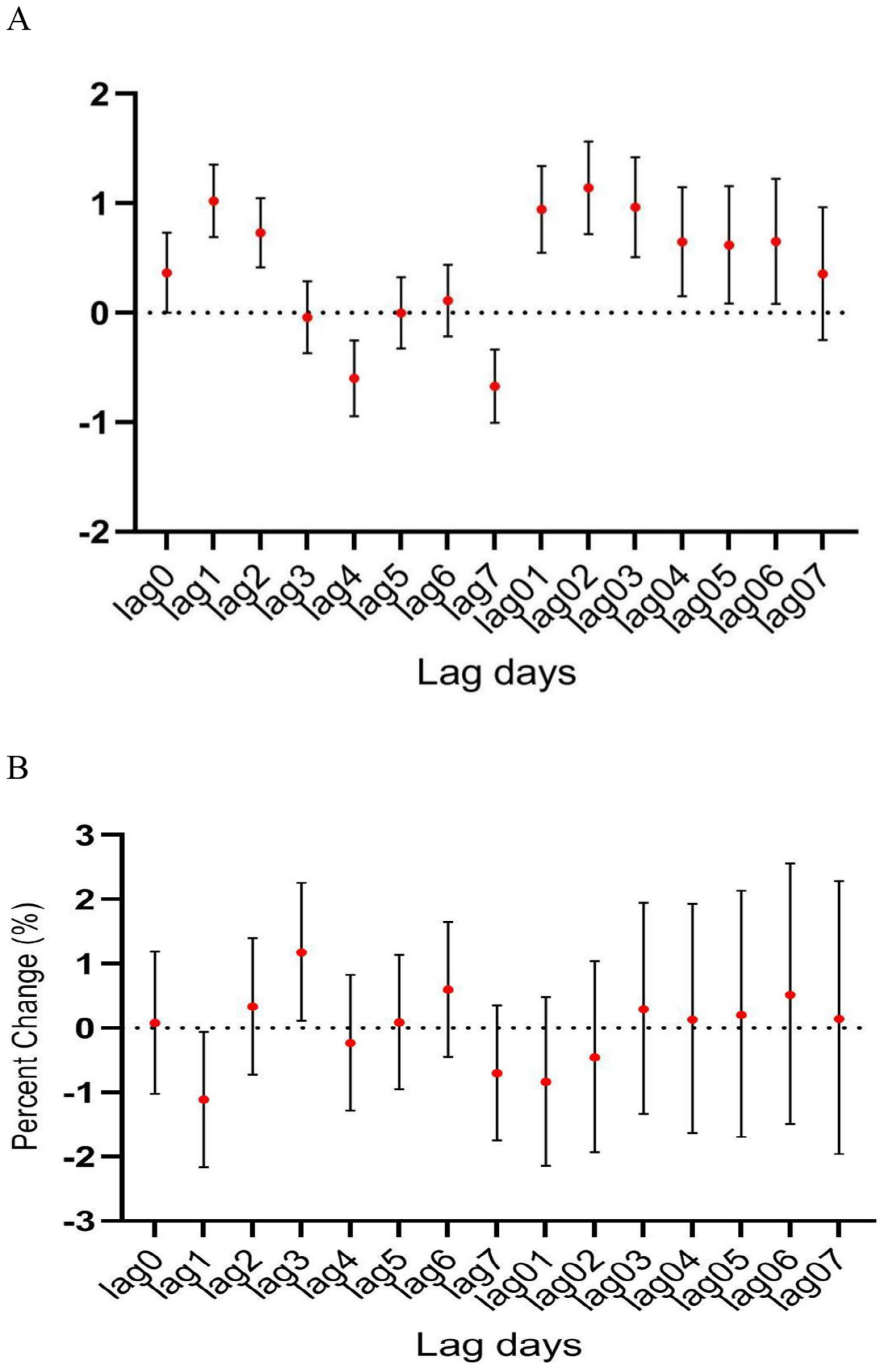
The relationship between atmospheric CO concentration and hospitalization for mental and behavioral disorders in **(A)** warm and **(B)** cold seasons.

In age-specific analyses, the association was more significant among patients aged ≥45 years than among those younger than 45 years (Figure 4). Interestingly, the association between CO level and hospitalization number was more significant in warm than in cold seasons, despite the number of hospitalization cases being more in cold than in warm seasons (Figure 5). In the two pollutant models, after adjustment for nitric oxide and sulfur dioxide, the correlation of CO level with hospitalization number was still statistically significant (Table 2).

**Table 2:** Analysis of CO robustness using two-pollutant model.

Two-pollutant models		ER (95%CI)
CO	-	
	PM2.5	0.520 (−0.102,1.146)
	PM10	0.526 (−0.001, 1.056)
	SO2	1.694 (1.307, 2.083)
	NO2	1.290 (0.952, 1.630)
	O3	0.347 (−0.189,0.885)

### Blood MAPK3 mRNA in patients with depression

**Figure 6:**
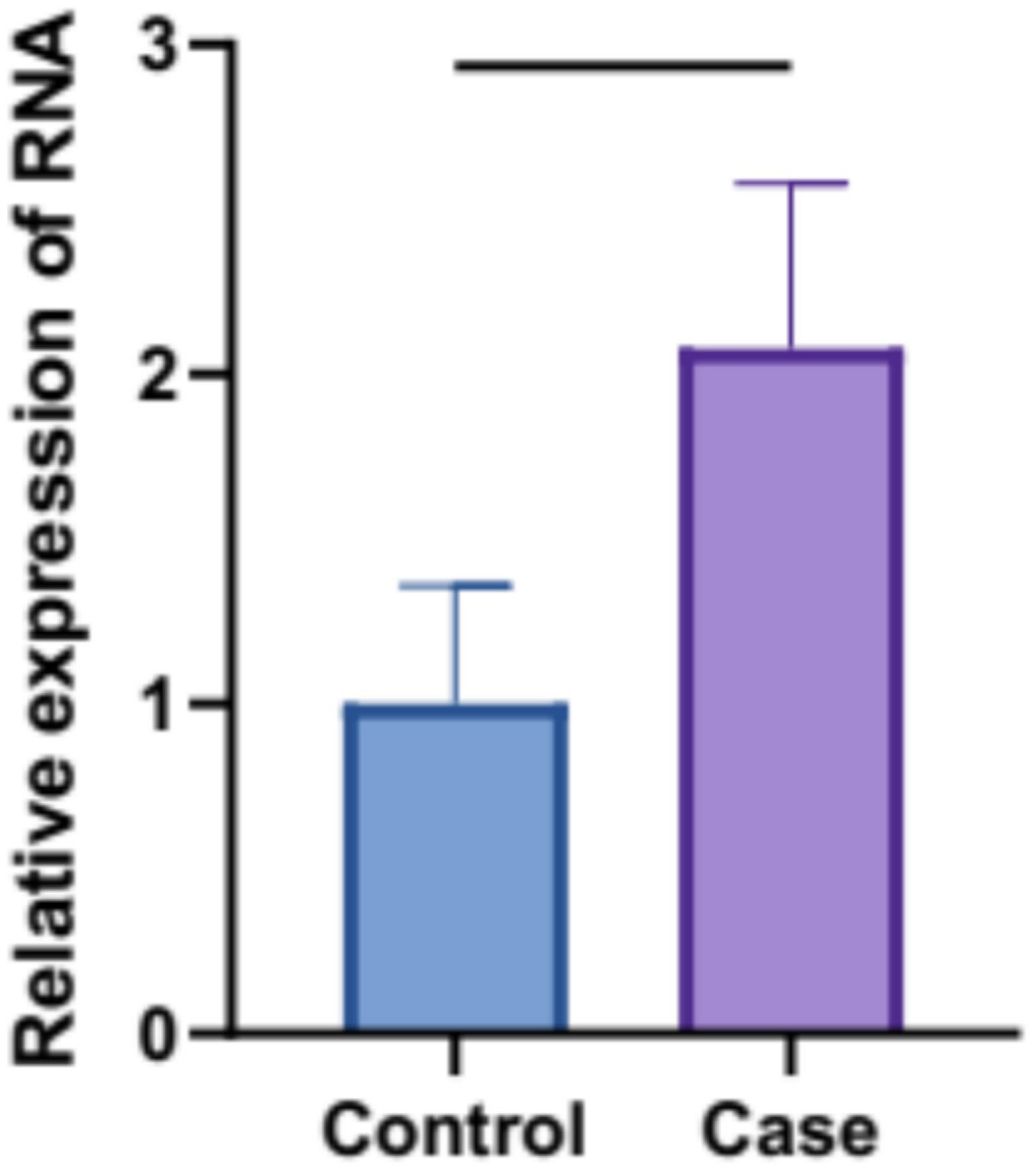
Blood MAPK3 transcript levels in patients with depression were determined using real-time PCR (*p* < 0.05).

Blood levels of MAPK3 mRNA in patients with depression (i.e., Case) were found significantly elevated by nearly 2 folds compared to those in healthy individuals (i.e., Control) (*p* < 0.05) (Figure 6). These results indicated the functional role of MAPK3 in the pathogenesis of depression, which would be validated in mouse model of CO-induced depression.

### Effect of MAPK3 inhibition on mouse model of CO-induced depression

In the sucrose preference test, the CO group mice showed a significant reduction in the percentage of sucrose preference compared to the Control group (*p* < 0.0001). However, these effects were largely reversed after PD98059 injection, with a significant increase in the sucrose preference percentage observed relative to the CO group. The Control + PD98059 group did not show a substantial difference from the levels observed in the Control group, with no significant statistical differences (Table 3).

**Table 3:** Data of sucrose preference test for model mice.

Group	Sucrose preference percentage (%)			
	Control	Control + PD98059	CO	CO + PD98059
Mean ± S.D.	77.66 ± 6.55	78.06 ± 5.51	54.20 ± 3.28	64.57 ± 6.62

**Figure 7:**
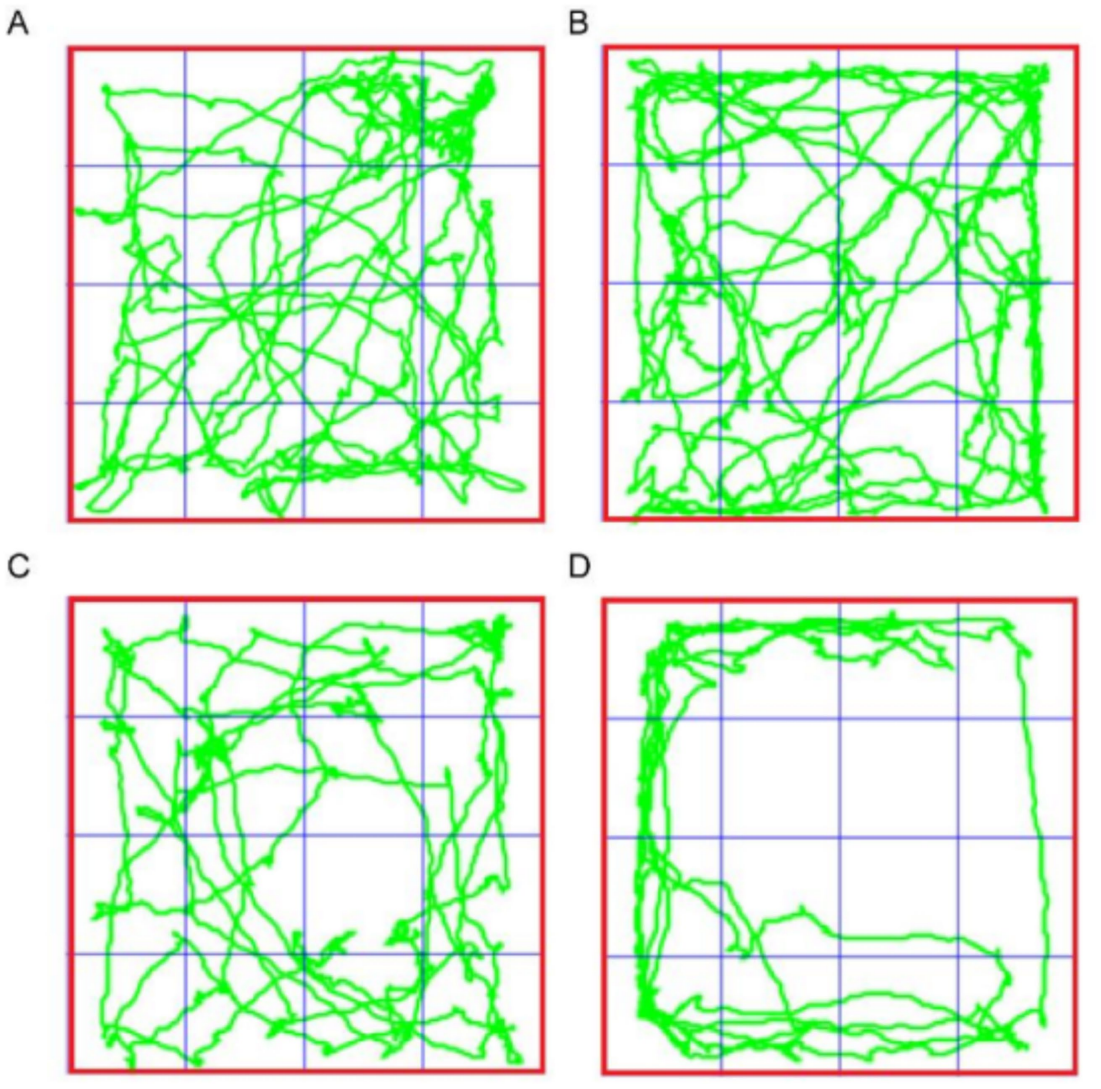
Open field test for mice of Control **(A)**, Control + PD98059 **(B)**, CO + PD98059 **(C)** and CO **(D)**.

In open field test, 24 h after PD98059 and saline injection, the total distance moved by the CO-exposed group of mice was significantly reduced compared to the Control group (*p* < 0.0001), indicating that CO exposure negatively impacted the mice’s motor ability or exploratory behavior. Notably, the total distance moved by the CO + PD98059 group significantly increased compared to the CO group (*p* < 0.05). In terms of exercise time, the CO group showed a significant reduction compared to the Control group, while the time spent immobile correspondingly increased, reflecting increased anxiety behaviors in the CO-exposed mice. The exercise time of the CO + PD98059 group showed recovery, with reduced immobility time, indicating that PD98059 injection effectively alleviated anxiety behaviors induced by CO exposure (*p* < 0.05). The number of times the CO group mice entered the central area (7.8 ± 5.93 times) was significantly less than that of the Control group (12.8 ± 10.62 times, *p* < 0.0001), suggesting that CO exposure reduced exploration behavior, possibly accompanied by increased anxiety-like symptoms. There was no significant difference in the number of entries into the central area between the Control + PD98059 group and the Control group (11 ± 10.37 times), indicating that PD98059 alone did not affect the exploratory behavior of the mice. The CO + PD98059 group entered the central area (1.6 ± 2.07 times), which was significantly increased compared to the CO group (*p* < 0.05) (Table 4; Figure 7).

**Table 4:** Data of open field test for model mice.

Group	Total distance (cm)	Exercise time (s)	Immobility time (s)	Times of entering central area
Control	1964.84 ± 454.36	286.82 ± 9.98	13.18 ± 9.98	12.8 ± 10.62
Control + PD98059	1894.35 ± 215.27	284.82 ± 8.81	15.18 ± 8.81	11 ± 10.37
CO	722.64 ± 322.12	228.55 ± 20.58	71.454 ± 20.58	7.8 ± 5.93
CO + PD98059	1341.23 ± 86.52	255.51 ± 6.07	44.49 ± 6.07	1.6 ± 2.07

**Figure 8:**
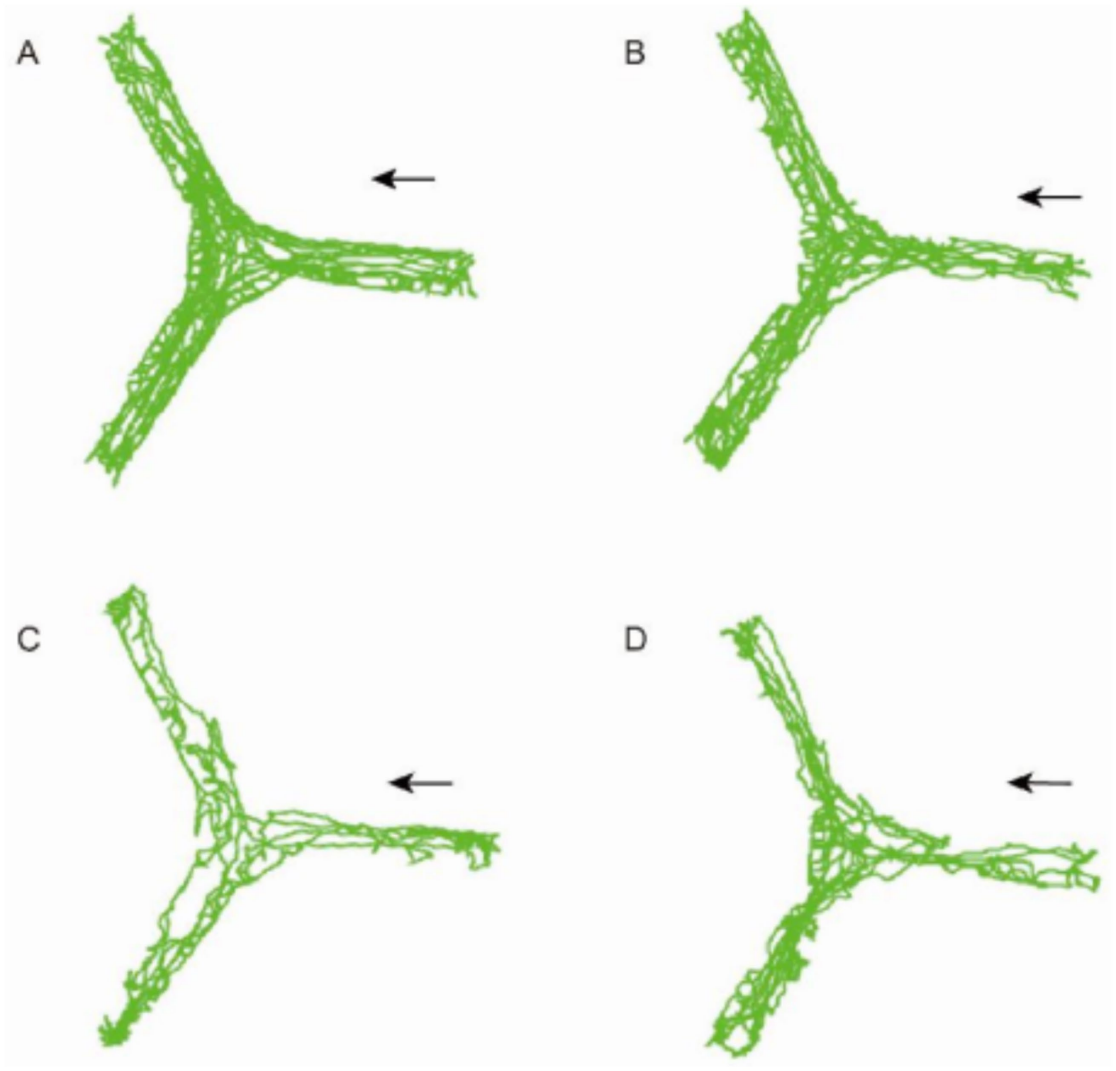
Y-maze field test for mice of Control **(A)**, Control + PD98059 **(B)**, CO + PD98059 **(C)** and CO **(D)**. The arrow indicates the direction in which the mouse is placed.

In the Y-maze test, the CO-exposed group of mice showed significant differences compared to the Control group, and the treatment with PD98059 affected these differences (Figure 8). The Control group and the Control + PD98059 group had higher scores for spontaneous alternation behavior, indicating good memory capability. The scores of the CO-exposed group significantly decreased (*p* < 0.01), while the scores of the CO + PD98059 group improved, suggesting that PD98059 has a certain ameliorative effect on CO-induced brain damage.

The results of the Y-maze experiment indicate that CO exposure negatively impacts the spatial working memory of mice, while the treatment with PD98059 can partially reverse these effects. PD98059 may improve CO-induced cognitive dysfunction by inhibiting the MAPK3 signaling pathway (Table 5).

**Table 5:** Data of Y-maze test for model mice.

Group	Total number of arm entries	Number of alternating entries	Number of entries	Spontaneous alternation behavior (%)
Control	24.4 ± 18.58	13.6 ± 7.83	22.4 ± 18.58	67.95
Control + PD98059	27.25 ± 9.18	16.75 ± 5.06	25.25 ± 9.18	67.65
CO	19.5 ± 9.68	8 ± 5.03	17.5 ± 9.68	41.38
CO + PD98059	30.75 ± 15.9	17 ± 7.87	28.75 ± 15.9	61.83

### Effect of MAPK3 inhibition on mouse model of CO-induced depression

**Figure 9:**
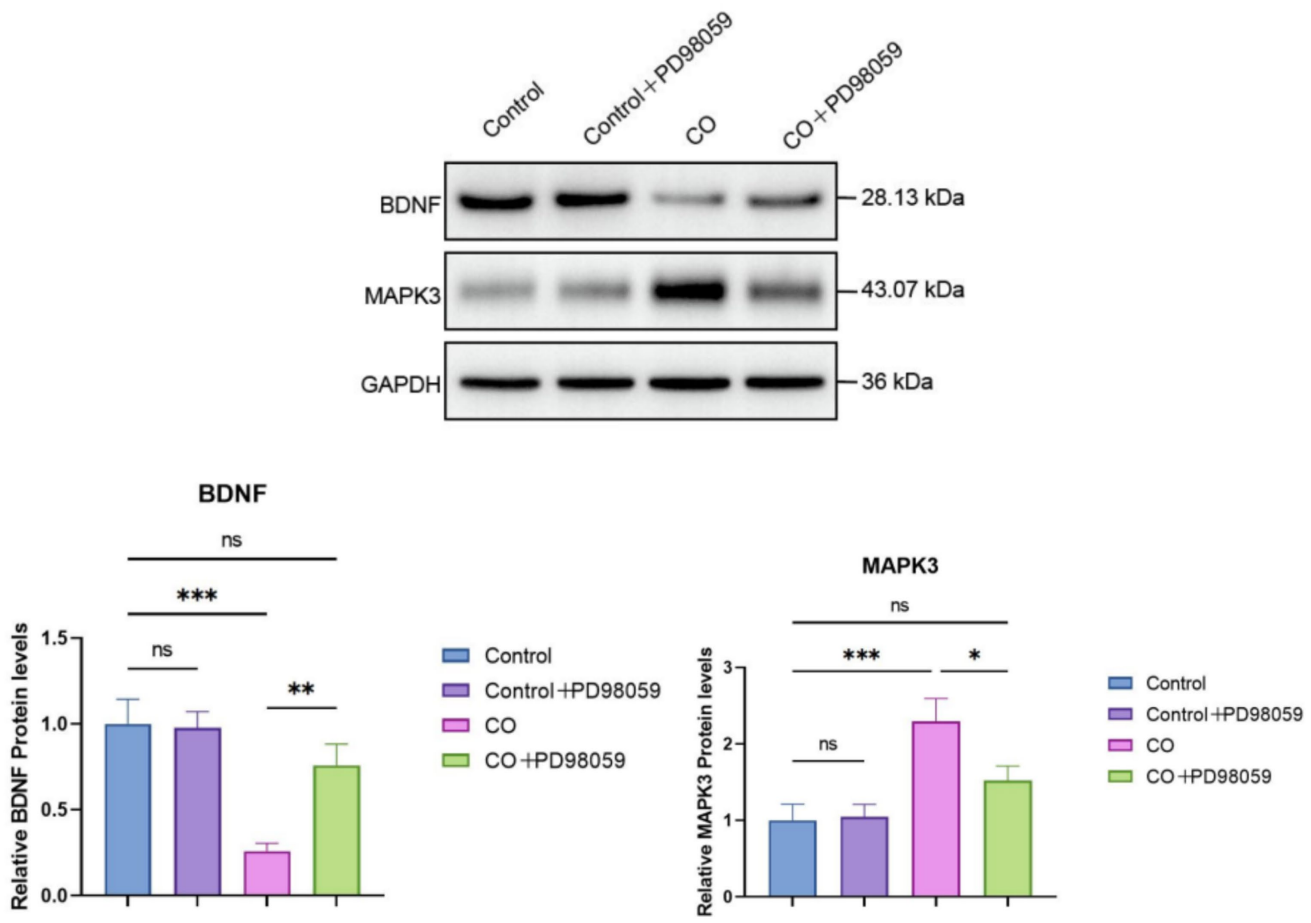
Protein levels of BDNF and MAPK3 in brain tissues of model mice were determined using western blotting. The band intensity was quantified and compared between treatment groups. *p < 0.05; **p < 0.01; ***p < 0.001; and ns, no significance.

Whether the inhibition of MAPK3 would modulate signaling was studied by determining the protein levels of BDNF and MAPK by western blotting (Figure 9). The expression levels of BDNF protein in the Control and Control + PD98059 groups were similar, both maintained at high levels. However, in the CO group, BDNF expression significantly decreased (*p* < 0.01), suggesting that CO exposure may inhibit BDNF expression. The expression level of BDNF in the CO + PD98059 group was significantly higher than that in the CO-exposed group (*p* < 0.01), although it remained lower than that in the Control group. The expression levels of MAPK3 protein in the Control and Control + PD98059 groups were relatively stable. In contrast, CO exposure significantly increased the expression level of MAPK3 (*p* < 0.001). In the CO + PD98059 treatment group, the expression level of MAPK3 was significantly lower than that in the CO-exposed group (*p* < 0.05), approaching the levels of the Control group.

## Discussion

The present study is one of the few to investigate the relationship between atmospheric pollutant CO and psychiatric hospitalization rates in developing countries. Based on analysis on the daily data from a major hospital in Shijiazhuang from 2014 to 2020, the results of this longitudinal study showed that short-time exposure to CO pollution would be significantly associated with an increased risk of hospitalization for mental and behavioral disorders. The effect of CO was found to be the most substantial on day lag02, and of importance, this association remained robust even after adjusting for other gaseous pollutants in the two-pollutant model. The transcript levels of MAPK3 in the blood samples of patients with depressive disorder were also found significantly elevated compared to healthy individuals. Inhibition of MAPK3 could alleviate the adverse effect of CO-induced depressive disorder in mice, strengthening the notion that MAPK3 and its associated signaling would be essential to mental disorders associated with CO pollution.

It is generally believed that particulate pollutants may cause various health problems, especially affecting respiratory and circulatory systems (Kan et al., 2008). In recent years, atmospheric particulate matter has also been considered as an important but unquantified risk factor affecting the mental system. There are indeed a few studies indicating the adverse effects of atmospheric pollutants (i.e., PM2.5, PM10, NO, etc.) on mental health (Calderón-Garcidueñas et al., 2015; Fan et al., 2020; Gu et al., 2020). There are few studies on its effect on mental health, despite environmental CO having been shown to have adverse effects on the circulatory and respiratory system (Liu et al., 2018; Orazzo et al., 2009). This study identified a significant association between CO and hospitalization for mental and behavioral disorders, of which the findings were in accordance with previous studies. A longitudinal study conducted in Shanghai indicated that the rate of hospitalization for mental disorders would be significantly associated with air pollutants (i.e., PM10, sulfur dioxide and CO) (Chen et al., 2018). It was also shown that increased ambient CO, nitrogen dioxide, and PM10 levels were associated with higher odds of depression among more than 120,000 Koreans (Shin et al., 2018).

Previous studies have shown that the elderly would be more susceptible to the effects of air pollution than young people (Song et al., 2018; Tong et al., 2016), which is basically consistent with the current findings, in which those aged more than 45 years were found more sensitive to the effects of CO. In terms of gender, this study showed that males would be more susceptible to the effects of air pollutants than females. The underlying mechanism has yet to be studied. An animal study indicated that the gender difference in neurotoxicity would be attributed to the differential expression of paraoxonase 2 between male and female (Costa et al., 2014). This study also found that the association between CO and hospitalization was stronger in warm than in cold seasons, which is consistent with a Canadian study (Szyszkowicz et al., 2009). It was believed that the strong association during warmer seasons might be due to greater outdoor activity so individuals would expose more to ambient air pollutants through ventilation (Zeger et al., 2000). There are also animal studies showing that the toxicity of air pollutants may increase under conditions of high temperature (Gordon et al., 2011). Additionally, heat stress has been found to promote neuroinflammation in mice, implicating high temperature may synergize with pollutants to promote neurodegeneration and subsequent sequelae of affective disorders (Lee et al., 2015). Interestingly, for other types of air pollutants, different seasonal patterns were shown. For example, Tong et al. found that the association between sulfur dioxide and nitrogen dioxide and psychiatric morbidity was stronger in cold season than in warm season in Tianjin (Tong et al., 2016). Lin et al. shown that there would be a close association between suicide rate and air pollution in cool season in Guangzhou (Lin et al., 2016). The seasonal pattern of the effect of air pollutants on mental health warrants further investigation, however, it is widely accepted that the differential patterns between pollutants would be due to complex factors such climatic conditions and exposure patterns in different geographic regions.

A complete picture about how air pollution would cause mental disorders has remained to be fully deciphered, however, studies have shown that air pollutants may alter normal brain functions and result in inflammatory responses and even pathological changes in brain tissues (Xu et al., 2016). The contribution of genetic and environmental factors to the development of mental disorders through activation of oxidative stress and inflammatory responses could be substantial. For example, an animal study revealed that exposure of the animals to particulate matters for 4 weeks could trigger depression-like response through upregulation of key neuroinflammatory cytokines like tumor necrosis factor-alpha and interleukin-1 beta (Hogan et al., 2015). Oxidative stress has been considered as one of the major pathways by which air pollutants cause damage to the cardiovascular and respiratory systems (Kelly, 2003). It is also evident that inhaled air pollutants can cause oxidative stress and system inflammation (Risom et al., 2005; Araujo, 2011), which would be able to affect psychological status (Ng et al., 2008; Hurley and Tizabi, 2013). The pathogenesis can be attributed to the damage of dopaminergic neurons by oxidative stress, which in turn depletes dopamine in the central nervous system, eventually resulting in depression and other mental disorders (Hasler, 2010; Block et al., 2004). Short-term exposure to CO pollution correlated with emergency department visits for mental disorders and related violent behavior (Tillakaratne et al., 2020), of which the underlying mechanism would also be due to the oxidative stress induced by CO.

The present study provided certain insights into how atmospheric CO level would affect hospitalization for mental disorders, however, since all CO measurements were done at fixed locations rather than individual measurements, the measurement of atmospheric CO level may have inevitable errors due to the fact that individual-level exposure can vary significantly based on factors such as time spent outdoors and personal activities. Besides, since data on more specific subtypes of mental disorders were not available for analysis, a comprehensive analysis on the CO-hospitalization relationship of the disorder subtypes was not possible. Analysis of short-term associations between specific air pollution and emergency room visits remains to be done as well. Due to her high level of urbanization, Shijiazhuang represents a model city for investigating how air pollution would affect mental health in China. Nevertheless, the findings of the present study require validations in other Chinese cities’ data, in which the repeated or planned hospitalization cases which are not inflicted with air pollution will be excluded. Other potential confounding variables like baseline health conditions, socioeconomic factors, and accessibility to healthcare service providers will also be studied.

This study also presented data to further support the important functional role of MAPK3 and its signaling pathway in CO-induced depression and mental disorders. MAPK3 transcript was found elevated in blood samples of patients with depression, while suppressing MAPK3 activity with an inhibitor could alleviate adverse effects in mouse model of CO-induced depression. These results might provide insights into the further development of MAPK3 inhibitors as potential therapeutics for treating CO-induced mental disorders in patients, although it is generally accepted that the CO exposure levels and conditions in the mouse model were highly controlled and may not reflect real-world human exposure.

This longitudinal study has demonstrated that short-term exposure to atmospheric CO would be associated with an increase in the number of hospitalizations for mental illness in Shijiazhuang city. The impact of CO was more obviously seen in the elderly, highlighting the urgent need to control CO emission to the atmosphere air pollution especially in China where the elderly population is growing rapidly. This study has also added to growing evidence for the adverse effects of air pollution on mental health and may help local policymakers develop and refine air pollution control measures, simultaneously, providing epidemiological perspectives for the future delineation of the causal relationship between particulate matter pollution and mental and behavioral disorders.

## Data Availability

The raw data supporting the conclusions of this article will be made available by the authors, without undue reservation.
